# Criterion validity of 8-item Morisky Medication Adherence Scale in patients with asthma

**DOI:** 10.1371/journal.pone.0187835

**Published:** 2017-11-30

**Authors:** Ana Janežič, Igor Locatelli, Mitja Kos

**Affiliations:** Chair of Social Pharmacy, Faculty of Pharmacy, University of Ljubljana, Ljubljana, Slovenia, EU; Universite de Bretagne Occidentale, FRANCE

## Abstract

The 8-item Morisky Medication Adherence Scale (MMAS-8) is reliable and valid in patients with hypertension, but to our knowledge validity has not been established for patients with asthma. The aim of the study was to determine the criterion validity of the MMAS-8 in patients with asthma. In the cross-sectional study patients older than 12 year were recruited when dispensed asthma medications in community pharmacies. Criterion validity of the scale was assessed through associations with asthma control and quality of life. Asthma control was assessed by the Asthma Control Test (ACT) and quality of life was evaluated by the Saint George Respiratory Questionnaire (SGRQ). A total of 208 patients (mean age 56 years, 59% female) were included in the study. Almost all patients were prescribed inhaled corticosteroids (96%). Asthma was not controlled in 37% of the patients and 22% experienced at least one exacerbation requiring emergency room visit, hospitalization or treatment with oral corticosteroid therapy in the previous year. The 8-item MMAS was significantly associated with asthma control and quality of life. Patients who scored 8 points, <8 to >6 points and ≤6 points on the scale were considered to have high, medium and low adherence, respectively. High, medium and low adherence was found in 53%, 23% and 24% of the patients, respectively. As adherence improved from low to medium or from medium to high, the odds of asthma control increased by 1.7 times (OR 1.65, p = 0.027). Patients with high and medium adherence had SGRQ scores that were 6.1 and 5.3 points lower, respectively, compared with patients with low adherence. The MMAS-8 was found to be valid for assessing medication adherence and predicting health outcomes in patients with asthma.

## Introduction

Poor adherence to long-term therapies significantly undermines the effectiveness of treatment and represents an important issue in disease control in terms of both the patients’ quality of life and health economics [1]. For patients with asthma, medication non-adherence is associated with suboptimal asthma control as well as more frequent hospitalizations. For example, Williams et al. found that for each 25% increase in the proportion of time without inhaled corticosteroid medication, the rate of asthma-related hospitalization doubled (relative rate 2.01; 95% CI 1.06–3.79) [2]. Moreover, regular use of inhaled corticosteroids is associated with a decreased risk of death from asthma [3].

Tackling medication nonadherence is a difficult task, starting with the first step, which is detecting nonadherence in clinical practice. Several different ways exist for measuring adherence to medication regimens. Each approach has advantages and disadvantages, and no method is considered the gold standard. Direct approaches, such as observed therapy or measurement of concentrations of a drug or its metabolite in blood, are expensive and burdensome to the patient and the health care provider. Consequently, indirect methods such as pill count or use of validated questionnaires are more suitable for use in clinical practice [4]. One of the most commonly used questionnaires to assess medication adherence is the Morisky Medication Adherence Scale. This scale was initially developed to evaluate medication adherence in patients with hypertension, but it is now widely used in various other patient populations. With a sum of scores equalling 8, 6 to <8, or <6, patients can be categorized as having high, medium or low adherence to therapy, respectively. These cut-off values were established based on the association between adherence to medication and blood pressure control in patients with hypertension. Morisky et al. found that blood pressure was controlled in 56.7% of patients with high adherence compared with 44.8% and 32.8% of patients with medium and low adherence, respectively [5]. The 8-item MMAS is reliable and valid in patients with hypertension [5,6,7], but to our knowledge validity has not been established for patients with asthma. Criterion validity measures how well one measure predicts an outcome for another measure. Hence, if criterion validity of the scale is established, it enables the prediction of health outcomes in patients based on the adherence determined by the scale. The aim of this study was to determine the criterion validity of the 8-item MMAS in patients with asthma.

## Methods

### Study subjects

The study sample comprised patients who met the following inclusion criteria: (I) had been prescribed at least one controller medication to treat asthma, including inhaled glucocorticoid (IGC), fixed combination of IGC and long-acting β agonist (IGC/LABA), leukotriene modifiers or systemic glucocorticoids; (II) were undergoing regular asthma therapy and had received ≥ 2 prescriptions; (III) stated they have asthma; (IV) were aged 12 years or older; and (V) were able to understand and communicate in the Slovenian language.

The sample size was calculated based on the assumption of 59% adherence, which comes from a systematic review of the literature. A total of 119 patients were required for the study, taking a 15% relative margin of error for the adherence estimation into account.

### Study design

We performed a cross-sectional study in 16 community pharmacies across Slovenia in September 2014 and February 2015. Eligible patients were invited to participate by community pharmacists when asthma medication was being dispensed. Those who agreed to participate were directed to a researcher who provided information about the study and obtained written consent from each patient. During the introductory interview the researcher collected patients’ demographic characteristics as well as information about asthma treatment and exacerbations in the past year. After the introductory interview, patients self-completed questionnaires to evaluate medication adherence, asthma control and quality of life. Patients were included in the analysis based on the self-reported diagnosis of asthma. Information about asthma medication and other medications that had been dispensed was also obtained from the pharmacy claims data. Moreover, to validate the data from patients regarding diagnosis, asthma treatment and exacerbations in the past year, information was obtained from participants’ GPs.

### Evaluation of medication adherence and health outcomes

Medication adherence was assessed using the MMAS-8. The scale consists of eight questions, first seven items having a dichotomous answer (yes/no) that indicates adherent or non-adherent behaviour. For item 8, a patient can choose an answer on a 5-point Likert scale, expressing how often happens that a patient does not take his medications. MMAS-8 scores can range from 0 to 8 points. Cut-off values for categorizing patients as having a high, medium or low adherence rate were chosen based upon association with asthma control and quality of life. The original MMAS-8 was translated to Slovenian language through forward/backward translation and tested for content validity in a pilot study.

Health outcomes included asthma control, quality of life and asthma exacerbations in the past year. Asthma control was evaluated by patients’ self-completion of the Asthma Control Test (ACT). The ACT is a reliable, valid tool that helps physicians identify patients with uncontrolled asthma in a clinical setting [8]. The questionnaire assesses the frequency of asthma symptoms and patients’ own perception of asthma control during the previous 4 weeks. Based on the sum of scores, complete asthma control (25 points), well-controlled asthma (20–24 points) or no asthma control (≤19 points) is revealed. We wanted to distinguish between patients with and without asthma control, therefore we used a score of 19 or less as the cut-off [8].

Quality of life was assessed using the Saint George Respiratory Questionnaire (SGRQ), which specifically measures health-related quality of life in patients with asthma and other airway obstruction diseases. The questionnaire has been shown to be reliable and valid [9]. It contains 50 items, divided into two sections. The first section pertains to patients’ recollection of their symptoms during the past year, and the second section addresses the patients’ current status. The questionnaire produces three component scores for symptoms, activity and impacts of disease. A total score can also be calculated which summarises the impact of the disease on overall health status, which covers a range of factors affected by the presence of symptoms that limit daily activities and social functioning of the patient. The total score was used for the analysis. It can range from 0 to 100, with higher scores indicating a lower quality of life. The SGRQ was translated to Slovenian language through forward/backward translation. Content validity was confirmed in a pilot study.

Asthma exacerbation was defined as the patient having had at least 1 event that indicated significant worsening of asthma control, namely, emergency room visit, hospitalization or therapy with oral glucocorticoids. Data about exacerbations in the 12 months prior to study participation was obtained during the introductory interviews. Information about the exacerbation was also obtained from GPs, but patient-provided data were used for analyses because of possibly incomplete data in the medical records.

### Analysis

Criterion validity of the scale was assessed through associations with asthma control and quality of life. The association between medication adherence and asthma control was estimated using a logistic regression model with two categories of asthma control according to ACT score (controlled and uncontrolled). Additionally, the logistic regression model was adjusted for potential confounders including gender, age, living alone or sharing a household, education, smoking status, the number of concomitant medications for other diseases, possession of an asthma self-management plan, exacerbation in the past year and medication adherence (low, medium, high). The association between medication adherence and health-related quality of life was estimated using linear regression modelling with quality of life (SGRQ total score) as the dependent variable. The same independent variables as in the logistic regression model were included. The association between occurrence of asthma exacerbation and asthma control or quality of life was evaluated by Mann-Whitney test. All statistical analyses were performed using the statistical package IBM SPSS v 23.0. A p<0.05 was considered statistically significant.

### Ethics statement

Written informed consent was obtained from all participants. For patients under 18 years old, written consent was also given by their parents. The study was approved by the National Medical Ethics Committee of the Republic of Slovenia.

## Results

### Study participants

A total of 208 participants met the inclusion criteria. The mean (SD) age of the patients was 56 (19.6) years, and the majority were female (59%). The patient characteristics are presented in Table 1. Almost all patients were prescribed IGC (96%), the majority in fixed combination with LABA (59%). Most patients took two medications for asthma treatment. The most commonly prescribed regimen was fixed combination IGC/LABA (once or twice daily) plus reliever medication (pro re nata). Data on the asthma medications prescribed to patients are presented in Table 2.

**Table 1:** Patient characteristics.

**Gender** (N = 208)	58.7% female
**Age** (N = 208); mean (SD)	55.5 (19.6)
**Current smoker** (N = 207)	19.3%
**Educational level** (N = 208)	
Elementary school	27.4%
High school	57.2%
College/university	15.4%
**Living alone** (N = 207)	23.2%
**No. of concomitant medications for other diseases** (N = 205); median (range)	2 (0–10)
**No. of medications for asthma treatment** (N = 208); median (range)	2 (1–5)
**Duration of asthma therapy** (N = 207)	
˂1 year	10.6%
1–5 years	31.9%
6–10 years	21.3%
˃10 years	36.2%
**Possession of asthma self-management plan** (N = 204)	14.7%

**Table 2:** Prescribed medications for treatment of asthma.

Medication type	% of patients
**IGC**	96
IGC/LABA	59
IGC only	36
**Reliever medication**	85
Salbutamol	41
Fenoterol/ipratropium	45
**LABA**	62
IGC/LABA	59
LABA only	3
**Leukotriene modifiers**	20
**Long-acting anticholinergics**	6
**Theophylline**	1
**Other (e.g. antihistaminics)**	8

IGC, inhaled glucocorticoid; LABA, long-acting β agonist.

### Health outcomes

Asthma was partially controlled and completely controlled in 21% and 43% of the patients, respectively. More than a third (37%) of patients had uncontrolled asthma. The ACT score ranged between 6 and 25, with a mean (SD) of 19.7 (5.1). The SGRQ total score ranged between 0 and 87.9, with a mean (SD) of 33.3 (21.8). The highest scores were obtained in the domain of symptoms (mean 42.5, SD 21.9), followed by activity (mean 39.8, SD 27.3) and impacts (mean 26.8, SD 22.5). Exacerbation that required emergency room visit, hospitalization and treatment with oral corticosteroid therapy was experienced in 9%, 9% and 14% of the patients, respectively. At least one of these events was experienced by 22% of the patients in the past year. Patients with at least one exacerbation in the past year had significantly worse asthma control (Mann-Whitney test; p<0.0005) and quality of life (Mann-Whitney test; p<0.0005).

### Medication adherence

MMAS-8 scores ranged between 0.75 and 8 points, with a median of 8. Patients’ responses showed that the most common reason for non-adherence was forgetfulness, followed by skipping doses when feeling better and other reasons besides forgetfulness.

### Medication adherence in relation to health outcomes

**Fig 1:**
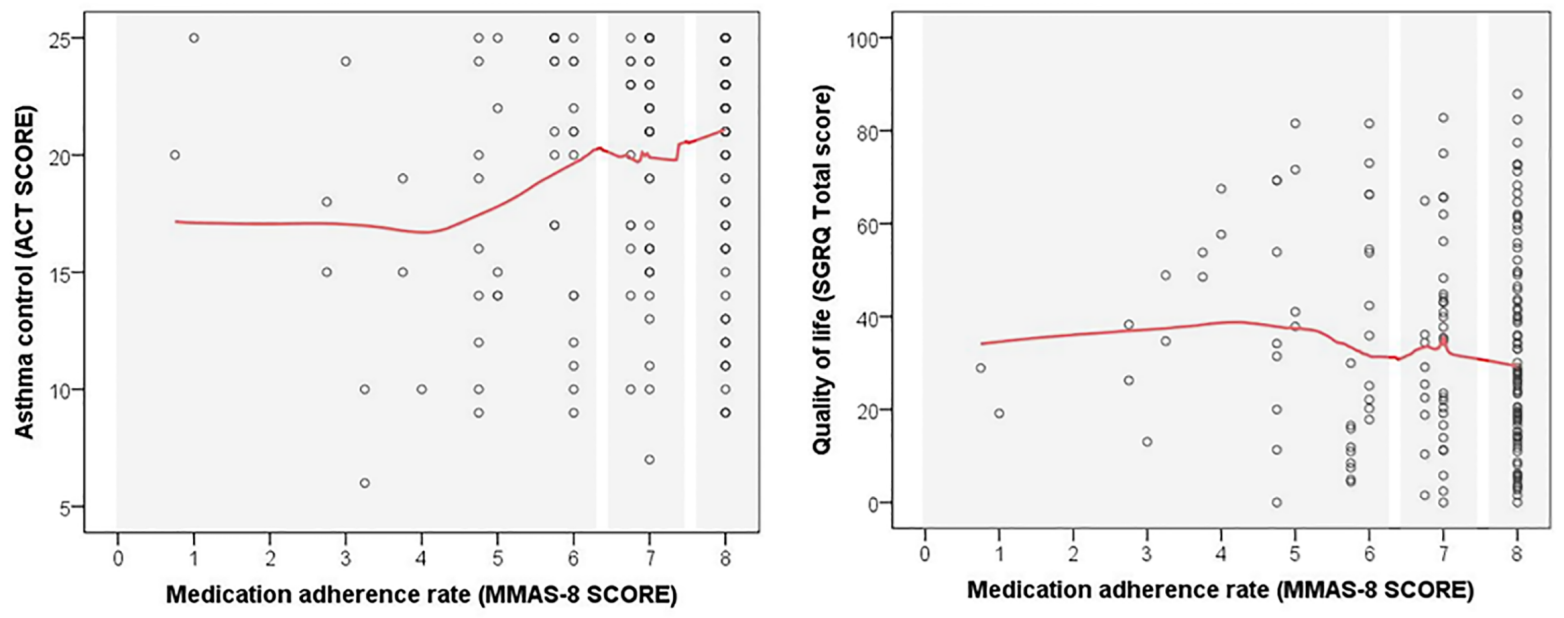
Scatter plot of asthma control (left panel) and quality of life (right pannel) in relation to medication adherence rate. LOESS curves are marked in red. MMAS-8, 8-item Morisky Medication Adherence Scale; SGRQ, Saint George Respiratory Questionnaire; ACT, Asthma Control Test. The use of the ©MMAS is protected by US Copyright laws. Permission for use is required. A licence agreement is available from Donald E. Morisky, MMAS Research LLC 14725 NE 20^th^ St. Bellevue WA 98007 or from dmorisky@gmail.com.

Cut-off values for categorizing patients as having low, medium or high adherence were chosen based on the relation to asthma control and quality of life (Fig 1). LOESS curves show a positive relationship between the medication adherence rate and the ACT score and quality of life (a higher SGRQ total score indicates poorer quality of life). Patients who scored 8 points on the MMAS (n = 110) were considered to have high adherence. The other patients were categorized into two groups based on the LOESS curves: patients who scored >6 and <8 points were considered to have medium adherence, and those who scored ≤6 points were regarded as having low adherence. High, medium and low adherence was found in 53%, 23% and 24% of patients, respectively.

The relationship between medication adherence and asthma control was evaluated using univariable logistic regression. Patients with high adherence had 2.3 times higher odds (95% CI for OR: 1.14–4.57, p = 0.02) for having controlled asthma compared to those with low adherence (reference), while patients with medium adherence had 1.3 times higher odds (95% CI for OR: 0.60–2.99, p = 0.47) compared to the reference. Nagelkerke R^2^ was 0.039. Based on these results, a linear relationship between categorized medication adherence and the occurrence of controlled asthma was assumed in subsequent analysis.

The multiple logistic regression model showed a statistically significant relationship between medication adherence and asthma control (Table 3). As adherence improved from low to medium or from medium to high, the odds of asthma control increased by 1.7 times (OR 1.65, p = 0.027). From among the potential confounders, education, smoking status and asthma exacerbation in the past year were found to have a statistically significant influence on asthma control. Patients who graduated from universities had more than 8 times higher odds (OR 8.65, p = 0.010) of asthma control compared to the patients with primary education. Current smokers had almost 3 times lower odds (OR 0.39, p = 0.034) of asthma control, and patients who experienced exacerbation in the past year had almost 6 times lower odds of asthma control (OR 0.18, p<0.0005) compared with those without asthma exacerbation. The logistic regression model with coefficients is shown in Table 3. The model significantly (p<0.0005) predicted asthma control with a 33.0% variance explained as estimated by Nagelkerke R^2^.

**Table 3:** Logistic regression model to predict asthma control.

Variable	Odds ratio	95% CI	p-value
Gender (female vs. male)	0.58	0.28–1.22	0.151
Age (years)	0.99	0.97–1.01	0.341
Sharing a household	0.79	0.32–1.91	0.594
Education (high school vs. primary school)	1.04	0.49–2.21	0.914
**Education (university vs. primary school)**	**8.65**	**1.69–44.31**	**0.010**
Number of concomitant medications	0.91	0.77–1.07	0.257
**Current smoker**	**0.39**	**0.16–0.93**	**0.034**
Possession of asthma self-management plan	0.98	0.37–2.58	0.966
**Asthma exacerbation**	**0.18**	**0.08–0.40**	**<0.0005**
**Medication adherence (3 categories-linear)**	**1.65**	**1.06–2.56**	**0.027**

The linear regression model showed medication adherence to be significantly associated with quality of life (Table 4). Patients with high and medium adherence had SGRQ scores that were 6.1 and 5.3 points lower, respectively, compared with patients with low adherence. Other factors that significantly influenced quality of life included age, number of concomitant medications for other chronic diseases, asthma exacerbation experienced in the past year and asthma control (ACT score). Older patients had a lower quality of life, with increase in SGRQ score of 0.2 points with each additional year. Similarly, with every medication for concomitant therapy of other diseases the SGRQ score increased by 2.4 points. Patients who experienced asthma exacerbation had a SGRQ score that was 8.1 points higher compared to those without exacerbation in the past year. Better asthma control was found to predict better quality of life; with every 1-point increase in ACT score, the SGRQ score would decrease by 2.5 points. The linear regression model (Table 4) was found to significantly (p<0.0005) predict quality of life, with 69.3% variance explained.

**Table 4:** Multiple linear regression model to predict quality of life.

Variable	Coefficient	95% CI	p-value
Gender (female vs. male)	−1.32	−5.65 to 3.02	0.549
**Age (years)**	**0.22**	**0.09 to 0.35**	**0.001**
Sharing a household	0.89	−4.41 to 6.19	0.740
Education (high school vs. primary school)	−2.23	−7.27 to 2.82	0.385
Education (university vs. primary school)	−2.76	−9.15 to 4.00	0.440
**Number of concomitant medications**	**2.38**	**1.34 to 3.43**	**<0.0005**
Current smoker	0.24	−6.74 to 7.22	0.947
Possession of asthma self-management plan	−0.86	−6.95 to 5.29	0.280
**Asthma exacerbation**	**8.10**	**2.96 to 13.2**	**0.002**
**Medication adherence (medium vs. low)**	**−6.06**	**−12.0 to −0.13**	**0.045**
**Medication adherence (high vs. low)**	**−5.29**	**−10.5 to −0.04**	**0.048**
**Asthma control (ACT score)**	**−2.49**	**−2.92 to −2.92**	**<0.0005**

## Discussion

To our knowledge this study is the first to determine criterion validity of the MMAS-8 in patients with asthma. The relationship between medication adherence and health outcomes might differ for asthma compared with hypertension or other chronic health conditions because of the specifics of the disease and the required therapy. Only if a tool for measuring adherence is validated can it be regarded as useful in clinical practice for specific patient populations. The cut-off values for defining high, medium and low adherence rates in this study differed from previous studies. In contrast to previous studies, the cut-off values in the current study were based on the association between adherence and asthma control as well as quality of life in patients with asthma. In contrast with Morisky’s cut-off values, a score of 6 points was assigned to the low adherence rate category because these participants were more similar to those with low, rather than medium adherence rates in terms of ACT and SGRQ scores.

A majority of patients (76%) were found to have a high or medium adherence rate, while a quarter did not adhere to their asthma therapy. Data on medication adherence in asthma patients as assessed by MMAS-8 is scarce in the international literature. Moreover, in studies conducted so far, cut-off values that were previously established for patients with hypertension were used. The proportion of adherent patients found in the present study is comparable to a study from Turkey (74%) [10]. However, lower proportions of adherent patients were found in a multicentric study conducted in Asia (53%) [11] and in a study including underserved rural community patients in the United States (44%) [12].

The quality of life of asthma patients in this study was comparable to that reported by other studies [13]. As in study by Joshi et al. [13], the highest scores were obtained in the domain of symptoms, which indicates the great impact that symptoms have on quality of life for asthma patients. This domain was followed by limitations in performing activities, impact of asthma on social functioning and psychological disturbances arising from airway disease. Based on a 1-year study of nedocromil for moderate asthma, mean changes in SGRQ total score of 4, 8 and 12 points are associated with slight, moderate and very good efficacy of treatment. Therefore, the minimal clinically important difference in SGRQ score is considered to be 4 points [14]. The results of the linear regression show that university education and medication adherence decrease SGRQ score by more than 8 points, while asthma exacerbation in the past year predicts an increase in SGRQ score of more than 12 points. Taking just two additional medications to treat comorbidities results in more than a minimally important increase in SGRQ. Hence, among other listed factors, medication adherence has a clinically important influence on the quality of life for patients with asthma.

The study showed that more than a third of the patients (37%) do not have asthma control. In a large cross-sectional survey in five European countries and the United States, a greater proportion of patients (43%) without asthma control was found [15]. In another large study of patients with asthma in seven European countries, almost half of all asthma patients (48%) reported a derived ACT score of <20 [16]. A proportion of asthma control similar to that found in our study was found by Nguyen et al., who showed that 36% of Vietnamese outpatients have no asthma control [17]. The majority of patients in our study and that of Nguyen et al. were using preventive medications. This preventive use may explain why the proportion of patients with uncontrolled asthma is lower than in previously mentioned European studies.

Better asthma control is associated with fewer exacerbations that may require emergency room visits and hospitalisations [18]. Twenty-two percent of all patients in our study experienced at least one exacerbation in the past year, defined as oral steroid treatment, asthma-related emergency room visit or hospital admission. Nine percent of all patients visited an emergency room and the same number were hospitalized. Patients in a European study who had poorer asthma control had more serious asthma-related events; 31% used unscheduled medical care and 13% were admitted to hospital [16].

Few patients with a very low MMAS-8 score were found to have a high ACT and a low SGRQ score. In some cases this pattern might be explained by “reverse” medication adherence. After achieving good disease control and quality of life, a patient may decide to adjust therapeutic regimen according to their own judgment, leading to poor medication adherence. Boland et al. found an indication of reversed causality in patients with chronic obstructive pulmonary disease (COPD) [19]. Improved quality of life during the first year was associated with reduced medication adherence during the second year, whereas no improvement in quality of life was associated with an increase in medication adherence [19]. Unexpected pattern can be observed with patients who gained 5.75 or 6.75 points by answering “once in a while” or “sometimes” instead of “never” on the last item about how often they forget to take their medication. They stand out with regard to asthma control and quality of life and would be expected to have a lower ACT score and a higher SGRQ score. The reason might be a less pronounced desirability bias in these cases compared with those of other participants in the study.

This study should be interpreted in light of its limitations. First, the study sample consisted of individuals with self-reported asthma. Data about diagnosis was also provided by patients’ GPs. We found a great agreement between patient-obtained and GP-obtained information regarding an asthma diagnosis. In 90% of the cases, patients’ self-reported asthma was confirmed by their medical records. The remaining patients had asthma-COPD overlap syndrome (5%), COPD (3%) or other respiratory condition (2%). We obtained data for 42% of the patients. Information from medical records was not available for the other participants because they did not approve access to their records or because of a lack of their GPs’ cooperation. All enrolled patients were included in the analysis. However, the relation between medication adherence and health outcomes might differ if patients have other diagnoses. Second, adherence can be overestimated when evaluated by self-report. Therefore, the result might overestimate the true medication adherence among asthma patients in Slovenia. We tried to minimize social desirability bias by administering the questionnaire to the patient in the presence of a researcher and not the pharmacist or a patient’s doctor. Data about symptoms and medication use were based on patient recall over a period of 1 month or 1 year, which could potentially introduce recall bias.

## Conclusions

The study shows that medication adherence is associated with asthma control as well as with quality of life in patients with asthma. The 8-item MMAS is valid for assessing medication adherence in patients with asthma. Adherence as assessed by the scale can predict health outcomes in patients with asthma.
